# Follow-up evaluation of children with birth weight less than or equal to 2,000 g

**DOI:** 10.1590/S1516-31802004000600003

**Published:** 2004-11-04

**Authors:** Marcia de Freitas, Arnaldo Siqueira, Conceição Aparecida de Mattos Segre

**Keywords:** Anthropometry, Newborn infant, Low birth weight infant, Very low birth weight infant, Fetal growth retardation, Antropometria, Recém-nascido de baixo peso, Recém-nascido de muito baixo peso, Retardo do crescimento fetal

## Abstract

**CONTEXT:**

During the first year of life, the growth process is highly vulnerable to several impairing factors that need to be understood.

**OBJECTIVE:**

To perform follow-up evaluation on newborns weighing less than or equal to 2,000 g in a population of low socioeconomic level.

**TYPE OF STUDY:**

Retrospective.

**SETTING:**

Hospital Maternidade Escola de Vila Nova Cachoeirinha, São Paulo, Brazil.

**METHODS:**

The study included 60 children born between March 1996 and January 1998, weighing less than or equal to 2,000 g. They were divided into three subgroups, according to birth weight and adequacy for gestational age. The factors studied were maternal variables, illnesses among the newborns, hospital admissions subsequent to discharge from the nursery, and the evolution of weight from birth until 12 months of life. Statistical analyses were performed through application of the Statistical Package for Social Sciences (SPSS) V.9.0 and Curve Expert 1.3 programs.

**RESULTS:**

Previous maternal diseases occurred in 38.6% of the pregnant women and intercurrent events occurred in 100%. The prevailing neonatal diseases were sepsis (30%) and hyaline membrane disease (25%). There were 404 visits on an outpatient basis: the most frequently diagnosed complaints related to respiratory diseases (26%). Among visits to specialists, 81.7% were to the neuropediatrician. A diagnosis of normality was made for 80% of all visits, for all specialties. For each of these groups, a growth curve was established. These were shown to be below the reference curve standards, with such differences least evident with regard to the children's corrected age.

**DISCUSSION:**

The severity of the newborns'conditions may be related to the high incidence of maternal diseases prior to pregnancy as well as intercurrent events during pregnancy. The differences in growth in relation to NCHS charts show that corrected age should be used as a parameter.

**CONCLUSIONS:**

Socioeconomic conditions, clinical/obstetric events and newborn diseases during the hospital stay had repercussions on these children's progress during their first year of life. Their growth profile was found to be very far from the reference standard, thus indicating a need for constant, differentiated assessment.

## RESUMO

**CONTEXTO:**

O processo de crescimento durante o primeiro ano de vida é altamente vulnerável a múltiplos fatores que podem prejudicá-lo, o que torna necessário seu conhecimento e controle rigoroso.

**OBJETIVO:**

Avaliar o acompanhamento de recém-nascidos com peso de nascimento ≤ 2.000 g, de uma populacao de periferia de grande cidade, durante o primeiro ano de vida.

**TIPO DE ESTUDO:**

Retrospectivo, a partir de dados secundários.

**LOCAL:**

Hospital Maternidade Escola de Vila Nova Cachoeirinha, São Paulo, Brasil.

**MÉTODOS:**

60 recém-nascidos com peso ≤ 2.000 g, divididos em trâs subgrupos conforme peso e adequação à idade gestacional, foram estudados de março de 1996 a janeiro de 1998. Variáveis maternas, variáveis relativas ao seguimento das crianças no primeiro ano de vida, às admissões após a alta hospitalar e relativas ao peso durante o primeiro ano de vida foram estudadas. As análises estatísticas utilizaram o programa SPSS (Statistical Package for Social Sciences, SPSS) V.9,0 e o Curve Expert 1.3.

**RESULTADOS:**

Doenças maternas prévias ocorreram em 38,6% e em 100% dos casos houve intercorrâncias durante a gravidez. Das doenças neonatais, as de maior prevalância foram sepse (30%) e doença de membranas hialinas (25%). No ambulatório realizaram-se 404 consultas e as doenças respiratórias corresponderam ao diagnóstico de maior freqüância (26%). Das consultas com especialistas, aquelas com o neuropediatra corresponderam a 81,7%. Em 80% das consultas em todas as especialidades fez-se o diagnóstico de normalidade. Foi construída uma curva de crescimento para cada grupo e feitas comparações com as curvas de referância do National Center for Health Statistics que se mostraram abaixo dos padrões das curvas de referância, diferença menos evidente para a idade corrigida das crianças.

**DISCUSSÃO:**

O serviço onde foi realizado este estudo é referância para gestantes de alto risco de baixo nível socioeconômico. A gravidade das condições dos recém-nascidos pode estar relacionada com a alta incidância de doenças maternas antes da gravidez e intercorrâncias durante a gestação. As diferenças no crescimento em relação aos padrões do NCHS mostram que a idade corrigida deveria ser usada como parâmetro de crescimento para recém-nascidos de baixo peso.

**CONCLUSÕES:**

Condições socioeconômicas, eventos clínico-obstétricos e afecções apresentadas pelos recém-nascidos durante a internação repercutiram sobre sua evolução no primeiro ano de vida e seu perfil de crescimento distanciou-se do padrão referen-cial indicando necessitarem de avaliação constante e diferenciada.

## INTRODUCTION

Significant improvements in fetal medicine have led to better knowledge of pregnancy physiology, fetal development and some disorders affecting the conceptus and the mother. The combination of preventive, diagnostic and therapeutic actions have allowed for better embryo evaluation and more precise care in relation to fetal health. The fetus is now considered to be a patient; a person enjoying all the rights of citizenship.^1,2^

All of these advances have led to significant reductions in the mortality rate among very low birth weight newborns. Not so long ago, the viability of such infants was still a matter for discussion. Such advances have enabled these small premature children to go to school and participate in family and social contexts, with good possibilities for playing a productive role in the future. Therefore, it is of paramount importance to care for their growth and development, i.e. the set of physical and intellectual changes a child will undergo before reaching adulthood.^3^

Infant growth monitoring is an essential healthcare action that is particularly important in relation to children born under adverse conditions. During the first year of life, the growth process is highly vulnerable to multiple impairing factors because of its intense nature, and thus requires rigorous control.^4^

A deeper understanding of high-risk newborns’ history of growth and development, as well as the diseases affecting them during the immediate neonatal period, requires supplementary healthcare services that complement those already rendered, and the continuity of such services. Survival is not the only aim in neonatal intensive care, but is rather the first step in these children's lives. Outpatient follow-up of these "technological survivals" has therefore become a great challenge, as this is a task involving various healthcare professionals and also the infants’ families.^3^

The aim of this study was to investigate some variables that have an influence on the development of newborns whose birth weight was less than 2,000 g, during their first year of life. The study was based on follow-up work carried out in a tertiary-level public hospital that is a referral center for high-risk pregnant women. The data gathered from these children were compared with the standards for children's growth of the National Center for Health Statistics (NCHS).^5^

## METHODS

This was a descriptive and retrospective study covering a population of children born between March 1996 and January 1998 that was followed up at the high-risk outpatient service of the hospital and maternity school of Vila Nova Cachoeirinha, São Paulo, Brazil, from July 1996 through June 1998. All children with birth weight of less than or equal to 2,000 g and gestational age of less than or equal to 36 weeks who were followed up during the first year of life and had made at least two medical visits were included. During this period, a total of 122 high-risk children were discharged from the hospital's neonatal intensive care unit and were referred for follow-up at the hospital's high-risk outpatient care facility. Those with congenital malformations or birth weight of over 2,000 g, and those that made fewer than two outpatient visits, were excluded from the sample. Thus, a final total of 60 children were included in the study.

### Procedures

Newborns with birth weight ≤ 2,000 g upon discharge from the nursery were referred for outpatient follow-up at the same maternity facilities and, if they were within the inclusion criteria, they were allocated to one of the following three groups:

Group I – children with birth weight < 1,500g, ranked as adequate for gestational age.
Group II – children with birth weight < 1,500 g, ranked as small for gestational age.
Group III – children with birth weight between 1,500 g and 2,000 g, ranked as adequate for gestational age.

The first visit was set up for one week after hospital discharge and the children were to visit the hospital outpatient care facility every two months during their first year of life in order to consult with the pediatrician and the multidisciplinary team.

At each visit, the infant was weighed while naked and in dorsal decubitus, on a balance with a minimum capacity of 250 g, maximum capacity of 15 kg and divisions of 10 g. The weights were expressed in grams. Other anthropometric measurements were also taken, although not included in the evaluation. The study also encompassed both the corrected and chronological ages.

A behavioral evaluation was also carried out in the maternity ward by a multidisciplinary team, but these data were not included in this study. The nutritional follow-up was supervised by a nutrition specialist within the multidisciplinary team. Whenever recommended, the infant was referred to a specialist, not necessarily located within the institution.

### Data sources

The data were collected with the help of two standardized forms: the hospital admission form, containing both the mother's data and the newborn's data from birth until nursery discharge, and the outpatient follow-up form.

### Study variables

The variables studied were subdivided and grouped into:

- maternal variables;
- variables relating to the infant during the first year of life;
- variables relating to admissions after nursery discharge;
- variables relating to weight progression from birth until 12 months of life.

### Statistical analysis

The information gathered was placed in a databank using the Access software and the reports were exported in Word or Excel format. The statistical analyses were processed via the Statistical Package for Social Sciences (SPSS) version 9.0^6^ and the Curve Expert 1.3 program.

Comparisons between pairs of quantitative variables were made via the Student t test if they followed a normal distribution. Otherwise, the Mann-Whitney non-parametric test was applied.

The application of the chi-squared test and Fisher's exact test allowed analysis to be performed on the associations among the three groups and the different variables, or between the variables, regardless of the group.

Variance analysis was applied to the data repeatedly, to study weight progression during the first year of life, by comparing groups and genders. The regression equation that best described the growth from birth to 12 months of life in terms of weight was calculated for each group, with the aim of minimizing the influence of age variation on the results based on the observed data. Also, a further estimate of the best adjustment curve for weight data was made via regression analysis for each group. The quadratic regression model using the independent variables of month and month squared was the best in the three groups.

### Ethical considerations

The study was approved by the coordination department for teaching and research of the maternity hospital, under the provisions of Conselho Nacional de Saúde resolution no. 196, dated October 10, 1996.

The Committee for Ethics, Teaching and Research of the Faculty of Public Health, Universidade de São Paulo, approved the study.

## RESULTS

The mean age of the population of pregnant women was 24.02 years, with a range from 13 to 37 years. Of the whole population of mothers, 30% were adolescents; 28% were married and 60% had not completed their junior year of high school. Table 1 shows general characteristics of the population in the study.

**Table 1:** General characteristics of the population (n = 60) of newborns in a high-risk outpatient service in São Paulo

Characteristic	Mean	Standard deviation	Minimum value	Maximum value
Maternal age (years)	24.02	6.31	13	37
Gestational age (weeks)	30.63	2.35	27	36
Newborn's birth weight (g)	1,327.17	228.04	945	1,875
Newborn's hospital stay (days)	46.03	16.61	23	112

After variance analysis and multiple comparisons of gestational age among the three groups under study, no difference was found between groups II and III. Group I was found to differ both from group II (p = 0.008) and from group III (p = 0.002). The children's gestational age by group is shown in Table 2. No significant differences were noted in relation to maternal age, number of prenatal visits, schooling and type of delivery among the groups.

**Table 2:** Gestational age distribution according to minimum, mean, maximum and standard deviation values in the studied groups of newborns in a high-risk outpatient service in São Paulo

Gestational age per group	n	Mean	Standard deviation	Minimum	Maximum
Group I	32	29.59	1.67	27	34
Group II	17	31.56	2.72	27	36
Group III	11	32.18	2.14	29	35
**Total**	**60**	**30.63**	**2.35**	**27**	**36**

*Group I = children with birth weight < 1,500 g, ranked as adequate for gestational age; Group II = children with birth weight < 1,500 g, ranked as small for gestational age; Group III = children with birth weight between 1,500 g and 2,000 g, ranked as adequate for gestational age.*

Previous maternal diseases occurred in 38.6% and intercurrent events during pregnancy occurred in 100% of the patients. These resulted in 48.3% of the women being admitted to hospital during the gestational period, with an average 10.2 days of hospital stay. Two deaths occurred among the mothers (3.33%), which were secondary to hypertension, one of them also associated with an acute illness.

The main diseases noted during the newborns’ admissions to the neonatal intensive care unit were: sepsis, 30%; hyaline membrane disease, 25%; intracranial hemorrhage, 25%; bronchopulmonary dysplasia, 11%; and necrotizing enterocolitis, 9.0%. The statistical analysis carried out among the different groups for each one of these conditions showed significance via the chi-squared test only for hyaline membrane disease (χ^2^ = 6.963; p = 0.031) and sepsis (χ^2^ = 6.51; p = 0.039), which were significant associations for groups I and II. Two or more of these diseases could affect the same child; e.g. bronchopulmonary dysplasia occurred in 40% of the hyaline membrane disease cases, thus showing significant association between these entities (χ^2^ = 13.44; p = 0.001).

For the 60 children participating in the study, a total of 404 outpatient visits were made during the first year of life (mean of 7.3 visits per patient). The result from all the visits made was that respiratory diseases were found in 26% of the sample, blood and hematopoietic organ diseases in 13.4%, digestive system diseases in 12.4% and nervous system diseases in 10.9%. It was also noted that 93.3% of the children were assessed by some specialized professional category.

Table 3 shows the distribution among the various clinical specialties of children undergoing consultations. It can be seen that the highest percentage of consultations was with a neurologist. There were no significant associations among the various groups in relation to the number of consultations with a neurologist (χ^2^ = 1.147; p = 0.325). Considering all clinical specialties, a diagnosis of normality for corrected age was found in 80% of the visits.

**Table 3:** Distribution of visits by child according to type of specialty, for the groups studied in a high-risk outpatient service in São Paulo

Professional category	Group I		Group II		Group III		TOTAL	
	n	%	n	%	n	%	n	%
Neurology	23	71.9	15	88.2	11	100.0	49	81.7
Phonoaudiology	18	56.3	8	47.1	5	45.5	31	51.7
Ophthalmology	17	53.1	6	35.3	7	63.6	30	50.0
Physiotherapy	8	25.0	4	23.5		0.0	12	20.0
Cardiology	4	12.5	2	11.8	1	9.1	7	11.7
Otolaryngology	1	3.1	2	11.8	2	18.2	5	8.3
Pneumology	1	3.1	2	11.8	1	9.1	4	6.7
Hematology	0	0.0	3	17.6	0	0.0	3	5.0
Pediatric Surgery	2	6.3	0	0.0	0	0.0	2	3.3
Endocrinology	0	0.0	0	0.0	1	9.1	1	1.7

*Group I = children with birth weight < 1,500 g, ranked as adequate for gestational age; Group II = children with birth weight < 1,500 g, ranked as small for gestational age; Group III = children with birth weight between 1,500 g and 2,000 g, ranked as adequate for gestational age.*

The number of hospital admissions during the first year of life for each child ranged from 1 to 5 (mean of 1.7). Those affected by bronchopulmonary dysplasia and/or hyaline membrane disease during the neonatal period (60%) had the greatest number of hospital admissions.

**Figure 1:**
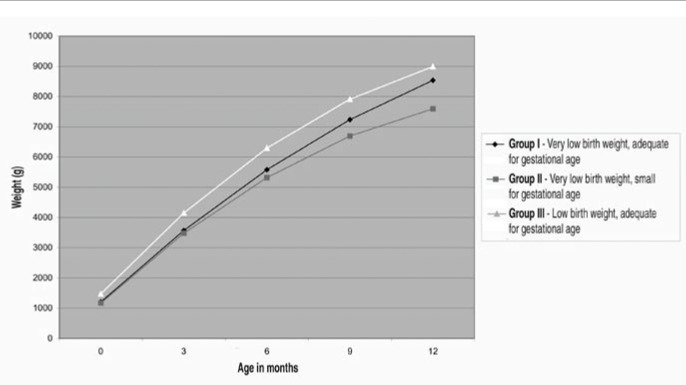
Growth curve for the three groups of children studied in a high-risk outpatient service in São Paulo.

For the purposes of growth assessment, growth curves were built for the first year of life, initially separated by gender. As these curves did not show significant differences between each other, they were then considered together, as illustrated in Figure 1 and Table 4, which shows the average weight gain for the three groups during these first 12 months of life, obtained by regressions.

**Table 4:** Mean birth weights (g)at ages 0, 3, 6, 9 and 12 months in groups I, II and III of children in a high-risk outpatient service in São Paulo

Months	Group I	Group II	Group III
0	1,203.54	1,168.04	1,671.22
3	3,569.10	3,478.88	4,148.36
6	5,579.88	5,319.92	6,294.14
9	7,235.88	6,691.16	7,908.56
12	8,537.10	7,592.60	8,991.62

*Group I = children with birth weight < 1,500 g, ranked as adequate for gestational age; Group II = children with birth weight < 1,500 g, ranked as small for gestational age; Group III = children with birth weight between 1,500 g and 2,000 g, ranked as adequate for gestational age.*

The comparison test between these curves showed significant differences between each other (F = 8.69; p < 0.001), as well as between the two regressions for groups I and II, i.e. for the children weighing less than 1,500 g (F = 3.25; p = 0.005).

**Figure 2:**
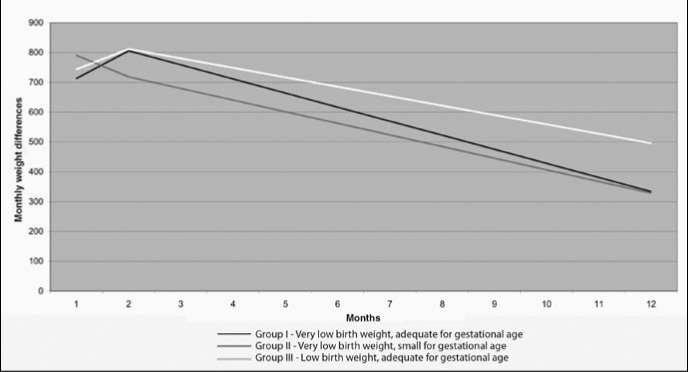
Weight gain distribution during the first year of life for the three groups of children followed up in an outpatient service in São Paulo.

Figure 2 and Table 5 show the mean weight gain distribution during the first year of life, highlighting greater weight gain in groups I and III up to the age of 3 months. In group II, the weight gain was greatest in the first month, gradually decreasing from then on.

**Table 5:** Monthly mean weight gain (g) in groups I, II and III of children in a high-risk outpatient service in São Paulo

Months		Groups	
	Group I	Group II	Group III
1	713.1	790.8	743.7
2	805.9	718.7	812.0
3	758.7	679.7	780.4
4	711.4	640.7	748.8
5	664.2	601.6	717.2
6	617.0	562.6	685.5
7	569.8	523.6	653.9
8	522.6	484.5	622.3
9	475.3	445.5	590.6
10	428.1	406.4	559.0
11	380.9	367.4	527.4
12	333.7	328.4	495.7

*Group I = children with birth weight < 1,500 g, ranked as adequate for gestational age; Group II = children with birth weight < 1,500 g, ranked as small for gestational age; Group III = children with birth weight between 1,500 g and 2,000 g, ranked as adequate for gestational age.*

**Figure 3:**
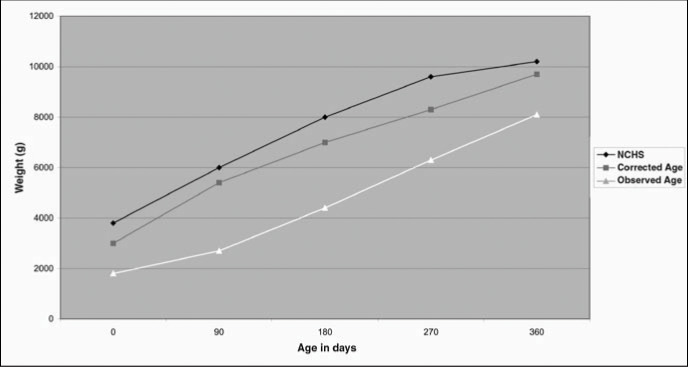
Comparison of weight of children followed up in an outpatient service in São Paulo with NCHS (National Center for Health Statistics) curves for male gender.

**Figure 4:**
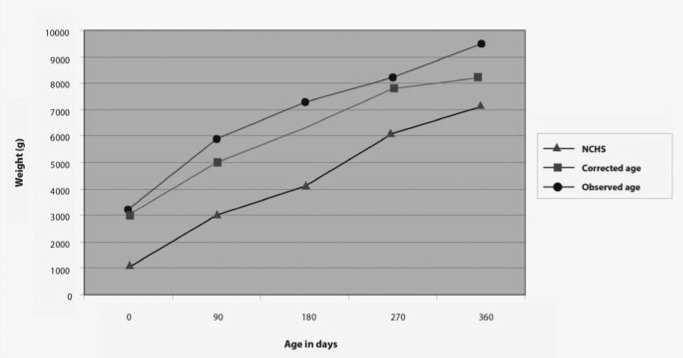
Comparison of weight of children followed up in an outpatient service in São Paulo with NCHS (National Center for Health Statistics) curves for female gender.

The gender-grouped growth curves for the present study population were also compared to the NCHS^5^ curves, as shown in Figures 3 and 4. At the age of 12 months old, the studied population showed less-than-expected weight performance for both genders, based on the NCHS^5^ reference standard, but the difference was found to be less evident when considering the corrected age.

## DISCUSSION

The hospital and maternity facilities where the present study was conducted are located on the periphery of a big city. They are known as a referral center for high-risk pregnant women from low socioeconomic classes, thus explaining the high frequency of risk factors among mothers and their children in this population.

A significant percentage of at-risk pregnant teenagers was found.^7-9^ Victora et al.^10^ have already emphasized that lower qualification levels and lower schooling are often associated with adverse effects on children during their first year of life. Gross et al.^11^ stated that premature infants are more affected in their progress by social conditions than by perinatal complications. As already found, simultaneous low education levels among mothers and high numbers of pregnant teenagers leads to speculation that, because of pregnancy, these girls had to withdraw from their formal education, either temporarily or definitively, thereby bringing problems to their children. A high incidence of maternal diseases prior to pregnancy was also found, as well as the fact that all these pregnant women suffered intercurrent events during pregnancy, thus contributing to the severity of these newborns’ conditions.

It is worth mentioning that there were two deaths among the pregnant women, leading to a maternal death coefficient of 3,333/100,000 live births, which is much higher than for the city of São Paulo: 58.34 and 58.27/100,000 live births for 1998 and 1999, respectively.^12^ It is extremely important to stress the severity of such data, despite the low number of pregnant women in the sample, especially because these deaths could have been prevented.

Among the outpatient diagnoses, respiratory diseases were found to be predominant, thus suggesting that the changes in respiratory dynamics that were noted in almost all of these children must have contributed to the increase in pulmonary secretions, thereby leading to the higher incidence of atelectasis and bronchopneumonia. During the first year of life, the number of hospital admissions was highest among newborns with bronchopulmonary dysplasia and/or hyaline membrane disease in the neonatal period, for both group I and group II. Furman et al.^13^ noted, in their analysis of the hospitalization and re-hospitalization spectra among children of very low birth weight with bronchopulmonary dysplasia during the first two years of life, that 50% of the newborns were admitted to a hospital in their first year of life, whereas this percentage dropped to 37% in the second year. In the present study, the number of children with bronchopulmonary dysplasia who were admitted to hospital during their first year of life was very close to the figures found by Furman et al.^13^ over a similar period of time.

Hematopoietic diseases were also frequent, and the most important of these was iron deficiency anemia. Such iron deficiency among premature infants is commonly caused by low iron reserves, blood withdrawal for laboratory tests and rapid growth, all of which are associated with the early withdrawal of breast feeding.^14,15^

The frequency of central nervous system diseases was also notable in the present study. Halpern et al.^16^ found, in their trials applying the Denver II test, that newborns with birth weight below 2,000 g had a risk of showing inadequate test results that was four times greater than for those with higher birth weight.

It is worth mentioning, however, that most of the children had normal diagnoses within the different clinical specialties. The performances of these children were also shown to be associated with their birth characteristics.

Relevant weight-related differences among the groups at the age cutoff points of three, six, nine and twelve months old were found. Regardless of the group studied, faster weight gain was noted during the first three months of life, although this was less pronounced for the newborns that were small for gestational age. Such weight recovery leads to the speculation that, for the majority of the selected newborns, their birth weight might have been adequate if they had reached a full-term age *in utero*.

Kashyap and Heird^17^ reported that it was impossible for newborns weighing less than 1,500 g to achieve growth compensation by the time of hospital discharge, i.e. to present the same fetal weight and body composition at the same gestational age. Ehrenkranz et al.^18^ concluded that newborns of gestational age of 24 to 29 weeks do not reach the fetal weight for the equivalent post-conception age. Lemons et al.^19^ reported that, in the light of the great advances in nutritional support, including minimum enteral nutrition introduction, the growth rate for newborns of very low birth weight could reach figures close to those of intrauterine growth, although not as fast as expected. The present study could not identify the rapid growth phase within the groups during the first year of life. However, it was noted that the highest weight gain occurred during the first months. A tendency for growth reduction in the final three months of the first year was also noted for all groups, including group III, the one with the highest birth weight.

It was also noted in relation to group III that, although these newborns showed small differences in weight gain in comparison with the other groups, the performances of these children in their monthly weight assessments, both on a quarterly and annual basis, always proved to be higher than those for the remaining groups. Group II, consisting of children with intrauterine growth restriction, presented the worst progress, while group I was found to present an intermediate behavior.

Bustos et al.,^20^ in an analysis of weights, lengths and cephalic perimeters for newborns of less than 1,500 g, concluded that a monthly weight gain of less than 750 g during the first three months of life constitutes the worst prognostic element. In the present study, it was found that all participating children had a weight gain of greater than 750 g during this period.

Jaruratanasirikul,^21^ in a longitudinal two-year study of low-weight newborns divided into six groups and compared with newborns of birth weight more than 2,500 g, concluded that all groups presented compensation growth during the first six months of life. For the newborns that were less than 1,500 g in weight and small for gestational age, this gain was significantly higher than for the other groups, but their weight was always below average.

Monset-Couchard and de Bethmann^22^ made a follow-up study of 166 newborns of birth weight less than 1,000 g and classified as small for gestational age, over an average period of nine years. They noted that, among newborns with symmetrical growth restriction, compensation growth occurred in 73%, while among newborns with asymmetrical growth restriction, there was compensation growth in 82%.

Hack and Fanaroff^23^ made a study comparing premature infants of birth weight of less than 750 g, with another group with a gestational age of 40 weeks that had similar features but had birth weights ranging from 750 to 1,449 g, as assessed at birth. These comparisons were made at the ages of eight months, twenty months and 6-7 years old, and it was noted that the group with birth weight of less than 750 g showed weights that were lower than those for the other groups at all the ages assessed.

When comparing the newborn growth curves in the present study with those from the NCHS^5^ standards, it was noted that the weight progress showed trends that differed from the NCHS^5^ reference values during the first year of life. For example, at the age of 12 months, the population studied showed weight performance that was below expectations in relation to the NCHS^5^ reference standards and also weight deficiency at the end of the period. However, the importance of the corrected age as a parameter for comparison with the reference values must be highlighted, since the difference was greater than in relation to chronological age.

These results lead to the conclusion that the socioeconomic conditions, clinical events (both obstetric and perinatal) and also the diseases identified in the newborns during their nursery stay influenced their progress during their first year of life. This was represented by respiratory, hematological, gastrointestinal and neurological diseases. However, most of the children were found to have a diagnosis of normality within the different clinical specialties.

The physical growth profile of children participating in this study was found to be very far from that of the children used for the NCHS^5^ reference standards. The newborns with birth weight of less than or equal to 2,000 g generally presented weight growth that was below the reference value average. This became more evident when considering the newborns that suffered intrauterine growth restriction, or the chronological age for all children. It seems consistent to suggest that growth curves should be constructed for premature infants, with the aim of making longitudinal assessments that are appropriate for the child's characteristics and thereby enabling early detection and correction of possible deviations as soon as they arise.
